# MSD-Net: Multi-scale dense convolutional neural network for photoacoustic image reconstruction with sparse data

**DOI:** 10.1016/j.pacs.2024.100679

**Published:** 2024-12-12

**Authors:** Liangjie Wang, Yi-Chao Meng, Yiming Qian

**Affiliations:** a Institute of Fiber Optics, Shanghai University, Shanghai 201800, China; b Key Laboratory of Specialty Fiber Optics and Optical Access Networks, Joint International Research Laboratory of Specialty Fiber Optics and Advanced Communication, Shanghai University, Shanghai 200444, China

**Keywords:** Biomedical imaging, Photoacoustic imaging, Deep learning, Convolutional networks

## Abstract

Photoacoustic imaging (PAI) is an emerging hybrid imaging technology that combines the advantages of optical and ultrasound imaging. Despite its excellent imaging capabilities, PAI still faces numerous challenges in clinical applications, particularly sparse spatial sampling and limited view detection. These limitations often result in severe streak artifacts and blurring when using standard methods to reconstruct images from incomplete data. In this work, we propose an improved convolutional neural network (CNN) architecture, called multi-scale dense UNet (MSD-Net), to correct artifacts in 2D photoacoustic tomography (PAT). MSD-Net exploits the advantages of multi-scale information fusion and dense connections to improve the performance of CNN. Experimental validation with both simulated and *in vivo* datasets demonstrates that our method achieves better reconstructions with improved speed.

## 1 Introduction

Photoacoustic imaging (PAI), as an innovative hybrid imaging technology, effectively combines the advantages of optics and acoustics to image biological tissues with high resolution and high contrast [1]. It has demonstrated broad application prospects in various biomedical fields, such as tumor detection [2], brain function imaging [3], and blood oxygen saturation measurement [4]. In photoacoustic tomography (PAT), the detection of acoustic pressure waves is achieved through detectors arranged around the sample. Detectors in PAT are typically arranged in various geometric shapes, such as spherical, cylindrical, and planar, to maximize signal acquisition and enhance imaging resolution. After acquiring the photoacoustic signals, image reconstruction is performed through inverse operations [5].

To obtain high-quality images, it is often necessary to use a large number of transducers for measurements. However, increasing the number of sensors results in higher production costs, greater hardware complexity, and increased computational demands on the device [6]. Therefore, it is necessary to limit the number of transducers under sparse-view conditions. Sparse-view photoacoustic imaging can effectively reduce system complexity and costs, as well as decrease the amount of data processing required, thereby accelerating imaging speed [7]. However, this approach may also result in decreased image quality during reconstruction, leading to artifacts and blurring. Hence, the influence of sparse-view conditions must be considered during the image reconstruction process [8]. If relying solely on standard methods such as Time Reversal (TR) [9], Delay and Sum (DAS) [10], Fourier beamforming [11], and Filtered Back-Projection (FBP) [12] for the reconstruction of sparse data, it often results in strong streaking artifacts in the reconstructed images, leading to a loss of spatial information [13]. Therefore, it is essential to adopt appropriate strategies under sparse-view conditions to enhance image quality and mitigate artifacts and information loss.

With the recent advancements in deep learning and the enhancement of hardware resources, deep learning-based PAI algorithms have seen unprecedented development [14], [15]. These algorithms can effectively supplement or replace traditional methods, providing higher-quality image reconstruction under sparse data conditions, thereby further advancing the application and development of PAI technology in the biomedical field. In PAI, there are three approaches to deep learning: image post-processing methods, direct image reconstruction methods, and hybrid processing methods. The direct image reconstruction method aims to directly convert ultrasound signals into photoacoustic images using a convolutional neural network (CNN) [16]. Guan et al. proposed the Pixel-DL method, which employs pixel-level interpolation based on the physics of photoacoustic wave propagation, followed by image reconstruction with a CNN [17]. In the hybrid processing method, Guo et al. proposed AS-Net, a network that takes raw photoacoustic signals and DAS-reconstructed images as inputs to generate high-quality PAT images [18], utilizing a multi-scale and multi feature fusion module to preserve more spatial information. In the post-processing method, photoacoustic images are initially obtained using standard techniques, and then imported into a designed deep learning network for enhancement. Guan et al. proposed a modified CNN architecture termed Fully Dense UNet (FD-Net) for PAT, which significantly reduced artifacts and produced high-quality PAT images in 2020 [19]. In 2022, Rajendran et al. proposed a deep learning based HD-UNet framework to address the low frame rate issue in circular PAT [20]. This framework successfully achieved high frame rate imaging of approximately 3 Hz while maintaining the high quality of photoacoustic reconstructed images. They also proposed UIU-Net, a novel deep learning method that utilizes photoacoustic images as reliable label images, thereby enhancing the accuracy of needle tracking during ultrasound-guided [21]. Direct reconstruction methods directly convert photoacoustic signals into images via CNN, which can efficiently process sparse data and generate high-quality images, but require a large amount of training data and have weak generalization ability [15]; hybrid processing methods combine the advantages of traditional reconstruction algorithms and deep learning, which can both improve image quality and recover details, but the computational complexity is high; post-processing methods are widely used, and they have been successfully applied in CT, MRI and other fields, especially effective in removing artifacts and enhancing image contrast [19].

In this work, we propose a modified CNN architecture, termed multi-scale dense UNet (MSD-Net), as a post-processing method to remove artifacts in 2D PAT images reconstructed from sparse data. The “UNet in UNet” structure (UIU Block) is incorporated into the encoding path to capture and integrate multi-scale information from the images, while dense connections are introduced in the decoding path. These dense connections reduce the learning of redundant features and enhance information flow, thereby improving the network's performance.

## 2 Methods

### 2.1 Architecture

**Fig. 1:**
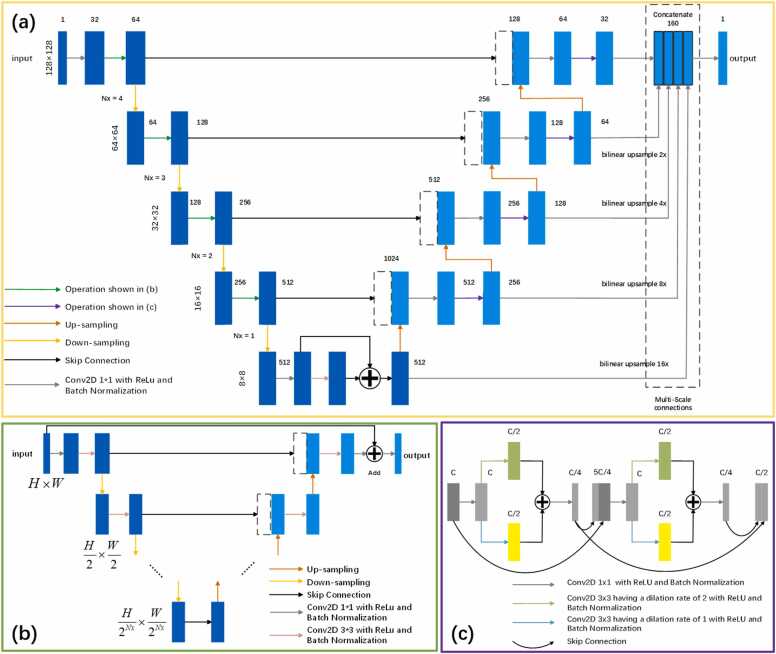
Network architecture for photoacoustic image reconstruction from sparse sampling. (a) The backbone architecture of MSD-Net. (b) Structure of the tiny U-Net (c) Structure of the dilated dense convolution block.

The U-Net is a CNN architecture proposed by Ronneberger et al. in 2015 [22]. It was originally designed for medical image segmentation and has been widely used in multiple fields, including semantic segmentation [23], image restoration [24], object detection [25], etc., due to its superior performance and flexibility. Especially in the field of photoacoustic imaging, the structural characteristics of U-Net make it perform excellently in image reconstruction and processing. In previous studies, U-Net formed a symmetrical U-shaped structure through ordinary convolution, up-sampling, down-sampling, and skip connections. However, it is difficult to capture enough useful information by relying solely on low-level, simple convolution operations, and it may not fully utilize the multi-scale feature information of the image [26]. In addition, in the decoder part of the U-Net, feature information may lose some useful details from the contraction path [27]. To address this issue, additional blocks such as dense blocks [19], residual blocks [19], [28], dilated convolution blocks [29], and multi-kernel size blocks [20] need to be added to traditional U-Net networks. In the latest research, researchers have proposed a U-Net in U-Net Network (UIU-Net) that integrates smaller U-Nets into larger U-Net backbones, achieving multi-level and multi-scale feature extraction [21]. Significant results have been achieved in infrared small target detection. However, using UIU modules in both the encoding and decoding paths can lead to increased layers and complexity, requiring a significant amount of memory and time. In order to improve the accuracy of photoacoustic reconstruction while reducing memory consumption, we propose an improved structure called MSD-Net based on UIU-Net, as shown in Fig. 1. The improved structure is built on the backbone of U-Net. In the extension path, we incorporate UIU blocks to capture multi-scale features, thereby enhancing the network's ability to perceive and learn from various levels of image. In the contraction path, we use dilated convolution kernels and dense block connections to obtain a larger receptive field while reducing parameters. Compared to UIU-Net, this approach is more concise and consumes less memory. We add multi-scale skip connections in the decoder output to combine low-level details with high-level semantics from feature maps of different scales, maximizing the utilization of feature maps at all scales and improving image reconstruction performance [30].

The MSD-Net network takes a 128 × 128 pixel image as input and consists of a contraction path, a bridging path, and an expansion path. The contraction path includes four coding layers, with each layer using UIU blocks for multi-feature extraction and 2x downsampling via max pooling. In the bridging path, residual blocks are used to maintain the continuity of information. The extended path consists of four decoding layers, each consisting of dense blocks with convolutional kernels of different dilation rates, enabling the network to learn features at different scales. After each decoding layer, bilinear upsampling is applied to restore the spatial resolution. To minimize information loss, the network uses a multiscale connectivity strategy after each decoding layer output, reducing the number of channels to 32 by convolution and using bilinear upsampling to restore the image size to its original 128 × 128 pixel. These outputs are then concatenated with the highest-resolution feature map to form a 160-channel feature map. Finally, the number of channels is reduced to 1 by 1 × 1 convolution to produce a 128 × 128-pixel synthetic image of the same size as the input image.

### 2.2 UIU block

The UIU block, as shown in Fig. 1(b) has a varying number of layers depending on the backbone level $N_{x}$. Specifically, when the backbone level $N_{x}$ = 4 and the input image size is 128 × 128, the UIU block consists of 4 layers. This design allows for deeper feature extraction and the capture of complex image information. The multiple layers enable the UIU block to capture low-level details and progressively extract more abstract high-level features, providing comprehensive information support for subsequent processing. In contrast, when the backbone level increases to $N_{x}$ = 1 and the input image size is reduced to 8 × 8, the number of layers in the UIU block is reduced to 1. This adjustment helps maintain computational efficiency and model simplicity. All UIU blocks across different levels are adjusted to an 8 × 8 feature map size after down-sampling. It optimizes the use of computational resources while ensuring that the model performs efficiently even with smaller image sizes. This design enables UIU block to flexibly adapt to different levels of needs. The high-level the UIU blocks can perform deep feature extraction to mine complex image information, while the low-level UIU modules maintain high computational efficiency and model simplicity. This enables MSD-Net to balance network depth and computational efficiency when processing different image sizes and feature extraction tasks, thereby improving overall performance and applicability.

### 2.3 Dense block

The dense block, as shown in Fig. 1(c). Dilated convolution is an advanced convolution technique that introduces spacing within the convolution process. This effectively enlarges the receptive field of the convolution kernel without significantly increasing the number of parameters or computational load. The design allows the network to capture contextual information from a broader area, enabling the extraction of richer multi-scale features in image processing. It is particularly beneficial for complex tasks such as semantic segmentation and object detection, where dilated convolution can significantly improve model performance.

In the decoding path, introducing dense blocks with multi-scale dilated convolutions can further amplify the network's representation capabilities. Specifically, in each expansion layer, convolution units with various kernel sizes, including 5 × 5 and 7 × 7 kernels derived from dilated operations on the original 3 × 3 kernels, are utilized. Compared to the FD-Net and HD-Net architectures, the 4-layer expanded convolutional layers are replaced with 2-layer convolutional layers to mitigate overfitting and reduce memory consumption. These multi-scale dilated convolution kernels can capture contextual information from different ranges, improving the network's ability to perceive both image details and overall structure. Batch normalization and the ReLU activation function are applied after each convolution block to accelerate network convergence and enhance the model's robustness to input perturbations.

### 2.4 Multi-scale connection

To learn hierarchical representations from full-size aggregated feature maps, a multi-scale connection module inspired by UNet 3 + was introduced, as illustrated in Fig. 1(a). The process begins with performing convolution operations on the output of each decoder stage to extract features. These convolved feature maps are then restored to a synthetic image size of 128 × 128 pixels using bilinear up-sampling, aligning with the input image size. Afterward, these up-sampled images undergo concatenation convolution operations, where multi-scale feature maps are concatenated to form a composite feature map. By integrating information from different scales, the concatenation convolution allows the feature map to maintain detail while possessing broader contextual information. Finally, the composite feature map is input into the Sigmoid function for nonlinear transformation, resulting in an image with a single channel. Through this approach, we successfully merged multi-scale features into a unified representation. This hierarchical representation not only retains the information from the original input image but also combines high-level features from various stages, resulting in a composite image with significantly enhanced structure and detail.

### 2.5 Network optimization and implementation

The proposed method is implemented in Python 3.7 using the PyTorch 1.10.1 framework and executed on an Nvidia Tesla V100 GPU. We use the MSE loss function to evaluate the error between the reconstructed image and the ground truth image. This MSE is defined as:

(1) $$\mathrm{MSE}=\frac{1}{n}\sum_{i=1}^{n}{\left(y-G\right)}^{2}$$

where n is the number of observations in the dataset, yis the reconstructed image, and G is the ground truth.

To evaluate the performance metrics of the reconstructed images, we use Peak Signal-to-Noise Ratio (PSNR) and Structural Similarity Index (SSIM) [31]. PSNR reflects the ratio between the mean square error of the original image and the noisy image (or reconstructed image) to the maximum possible value of the original image pixel values, measured in decibels (dB). The larger the PSNR value, the smaller the image distortion, indicating better image quality. PSNR is defined as:

(2) $${\mathrm{PSNR}=\mathrm{10log}}_{10}\frac{\max^{2}(y,G)}{\mathrm{MSE}}$$

where max(y,G) is the max value of y, G, MSE can be calculated by Eq. (1).

The SSIM is used to assess the similarity between two images by comparing their brightness, contrast, and structural information. SSIM is a number between 0 and 1, with a larger value indicating a smaller difference between the reconstructed image and the ground truth, thus reflecting better image quality. When two images are identical, SSIM = 1. SSIM is defined as

(3) $$\mathrm{SSIM}=\;\frac{\left(2\mu_{y}\mu_{G}+c_{1})(2\sigma_{yG}+c_{2}\right)}{\left(\mu_{y}^{2}+\mu_{G}^{2}+c_{1})(\sigma_{y}^{2}+\sigma_{G}^{2}+c_{2}\right)}$$

where $\mu_{y}$ and $\mu_{G}$ represent the mean values of the reconstructed image and the ground truth, $\sigma_{y}$ and $\sigma_{G}$ represent the variances of the reconstructed image and the ground truth, $\sigma_{yG}$ represents the covariance of the reconstructed image and the ground truth, and $c_{1}$ and $c_{2}$ are constants set to 0.01 and 0.03, respectively, to avoid division by zero.

Mean absolute error (MAE) evaluates the MAE between the target and speckle images. The MAE is defined as

(4) $$\mathrm{MAE}=\;\frac{1}{n}\sum_{i=1}^{n}\left|y-G\right|$$

where n is the number of observations in the dataset, y is the reconstructed image value, and G is the ground truth value.

To optimize the network, we used the Adam optimizer to update the model weights, with an initial learning rate of 0.005, a batch size of 4, and training for 150 epochs. We also implemented *k*-fold cross-validation (*k* = 10) to evaluate the network's performance. The best weights were obtained at epoch 84 of the third round, with an SSIM of 0.9838 and a PSNR of 39.82 dB.

## 3 Experiments

### 3.1 Simulated datasets

**Fig. 2:**
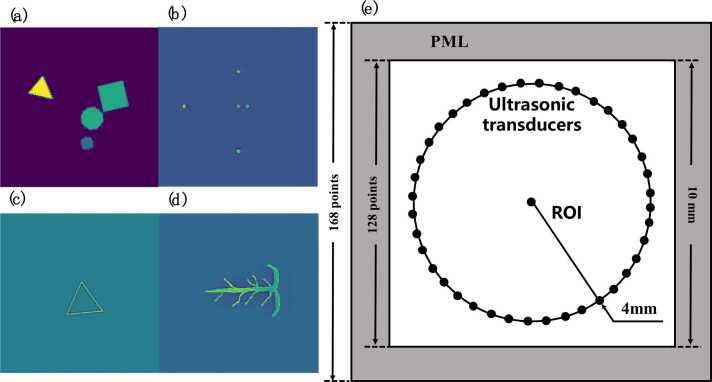
Samples of datasets and simulation geometry of the system. (a) Geometric phantom with random sizes and shapes. (b) Five-point sources numerical phantom. (c) Triangular numerical phantom. (d) Numerical blood vessel phantom. (e) Geometric schematic of the k-wave simulation in MATLAB. All sample images were generated through sparse sampling, using a circular array composed of 50 ultrasound transducers for data acquisition.

Deep learning is data-hungry, relying heavily on high-quality labeled images for effective training and performance improvement. However, photoacoustic imaging, as an emerging technology, is not as widely adopted as conventional medical imaging techniques like magnetic resonance imaging (MRI), computed tomography (CT), and ultrasound. The establishment of public datasets in the field of PAI is still in its infancy, presenting significant challenges in acquiring high-quality labeled images and expanding dataset sizes. Therefore, to address this issue, we use the k-Wave toolbox to generate simulated dataset [32]. The geometric schematic of the PAI scanning system simulated using the k-Wave toolbox is shown in Fig. 2. The imaging area, also known as the Region of Interest (ROI), is set as a 10 × 10 mm square area, divided into 128 × 128 grid points. An anisotropic absorbing boundary layer, also known as a Perfectly Matched Layer (PML), was used at the edge of the imaging area, with a width of 20 grid points. The propagation speed of ultrasound in the medium is set to 1500 m/s, which is a typical value commonly used in simulating biological tissues. The radius of the ultrasound transducer's circular array mask is set to 4 mm. The sampling angle is 2π. To simulate the sparsity of an actual array, we used 8–50 ultrasound transducers to simulate the sampling process of a circular array. At the same time, in the process of collecting sound pressure signals, we added Gaussian noise with a random signal-to-noise ratio of 40–60 dB to simulate the interference and noise that may exist in actual environments.

We generated several random objects within the imaging area, each with randomly selected center coordinates, radius (ranging from 5 to 10 pixels), size (ranging from 0 to 1), and rotation angle to simulate the variations in its position, size, intensity, and orientation in biological tissues. In terms of shape, we chose triangles, squares, and rectangles to simulate different biological tissues, as shown in Fig. 2(a). Photoacoustic images were reconstructed using the TR algorithm and standardized to a range of 0–1 before being used as input data for model training. Additionally, to expand the experimental dataset, we introduced the Multi_UST_PAT and Single_UST_PAT datasets (Rajendran et al., 2022) [20], which include five-point targets (made of pencil leads), triangular phantoms (made of horsehair), and numerical blood vessel phantoms, as shown in Fig. 2(b)-(d). After combining all the datasets, approximately 3000 images were used for the study, which were then divided into training, validation, and test sets at a ratio of 8:1:1.

### 3.2 in vivo and phantom dataset

**Fig. 3:**
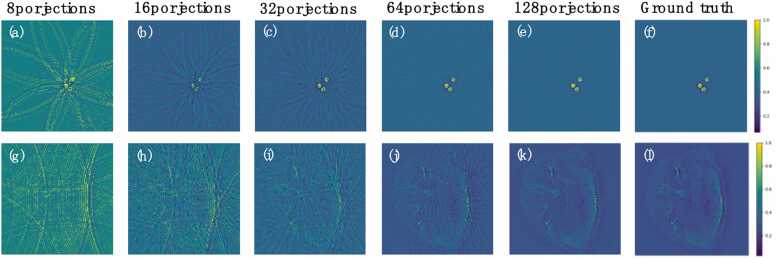
Samples of the *in vivo* dataset. (a)-(e) Photoacoustic tomography of agarose cylinders with different projections. (f) Full-sampled agar cylinder. (g)-(k) Photoacoustic tomography of whole-body mice with different projections. (l) Full-sampled whole-body mice.

The proposed MSD-Net was also tested on *in vivo* dataset. These data were obtained by the research team in Ref. [8] using a newly developed small animal imaging scanner to acquire images from live mice. During the scanning process, the torso region of each mouse was vertically scanned with a ring array at 0.5 mm intervals, covering the area from the shoulders to the lower abdomen, generating a total of 100 cross-sectional images. Additionally, we introduced phantom data, which were generated by imaging paper-printed absorbing targets embedded in a 16 mm diameter agar cylinder. Both the phantom data and the mice cross-sectional data were scanned with different numbers of probes, specifically including 16, 32, 64, and 128 sparse samples, as well as 512 projections as the ground truth，shown in Fig. 3.

## 4 Result

### 4.1 Performance comparison

We used *k*-fold cross-validation (*k* = 10) to evaluate the performance of our proposed MSD-Net and compared it with existing architectures such as UIU-Net, FD-Net, and U-Net. We also demonstrated its excellent performance through a series of quantitative metrics, including SSIM, PSNR, and MAE. The comparison results are shown in Table 1 and Table 2. MSD-Net, featuring 16.01 million parameters and 29.50 GFLOPs, exhibits higher computational efficiency and lower memory usage than UIU-Net, which has 50.57 million parameters and 40.96 GFLOPs. Tested on an RTX 2050 laptop, MSD-Net processed 100 images (128 ×128, 3-channel) at an average of 1.10 ms per image, 42.71 % faster than UIU-Net's 1.92 ms, as shown in Table 3.

**Table 1:** Performance comparison with different methods on simulated dataset (Mean ± Standard Deviation).

**Methods**	**SSIM**	**PSNR (dB)**	**MAE**
U-Net	0.9247 ± 0.0333	30.6583 ± 3.5025	0.3656 ± 0.3346
FD-Net	0.9542 ± 0.0661	34.5741 ± 4.5076	0.3432 ± 0.3215
UIU-Net	0.9773 ± 0.0111	37.3625 ± 4.1240	0.4291 ± 0.3614
MSD-Net	0.9838 ± 0.0105	39.8249 ± 5.7396	0.3309 ± 0.3811

**Table 2:** Performance comparison with different methods on *in vivo* dataset (Mean ± Standard Deviation).

**Methods**	**SSIM**	**PSNR (dB)**	**MAE**
U-Net	0.7317 ± 0.0654	28.6450 ± 2.5132	0.4217 ± 0.2334
FD-Net	0.7547 ± 0.0590	31.5558 ± 2.1679	0.4208 ± 0.2072
UIU-Net	0.7783 ± 0.0401	32.1055 ± 1.1376	0.6206 ± 0.0827
MSD-Net	0.8064 ± 0.0647	33.0620 ± 1.3930	0.3886 ± 0.1648

**Table 3:** Comparison of performance and computational efficiency of different methods.

**Methods**	**Params**	**FLOPs**	**Time per image (ms)**
U-Net	12.61 G	7.59 M	0.36
FD-Net	19.67 G	10.75 M	0.56
UIU-Net	40.96 G	50.57 M	1.92
MSD-Net	29.50 G	16.01 M	1.10

As shown in Table 1, MSD-Net performs excellently across all metrics. On the simulated dataset, MSD-Net achieves the highest SSIM of 0.9838, indicating the best structural similarity during reconstruction and excellent preservation of both global and local features. In comparison, UIU-Net's SSIM is 0.9773, which is good but slightly lower than MSD-Net's. For PSNR, MSD-Net ranks first with 39.8249 dB, demonstrating outstanding signal quality and noise suppression, with very low noise and high image quality. UIU-Net’s PSNR of 37.3625 dB is also high but lags behind MSD-Net, especially in maintaining image clarity and detail. Regarding the MAE metric, MSD-Net scores 0.3309, outperforming other models. Although UIU-Net performs well in SSIM and PSNR, its higher MAE of 0.4291 suggests some pixel-level precision issues. MSD-Net excels by maintaining structural integrity while effectively restoring details, resulting in the lowest MAE.

As presented in Table 2, MSD-Net remains the top-performing model. Despite the higher levels of noise and artifacts typically present in the *in vivo* and phantom dataset, MSD-Net maintains a leading position with an SSIM of 0.8064 and a PSNR of 33.0620 dB. This demonstrates that MSD-Net is capable of preserving structural integrity and signal quality in the more complex, noisy *in vivo* and phantom data, while also effectively restoring fine details, thereby ensuring high image quality. In contrast, UIU-Net performs slightly worse, with an SSIM of 0.7783 and a PSNR of 32.1055 dB, which are still high but not as good as MSD-Net. Furthermore, UIU-Net exhibits a slightly higher MAE of 0.6206, suggesting that it may prioritize the recovery of global structures over pixel-level detail.

### 4.2 Performance of MSD-net on simulated dataset

**Fig. 4:**
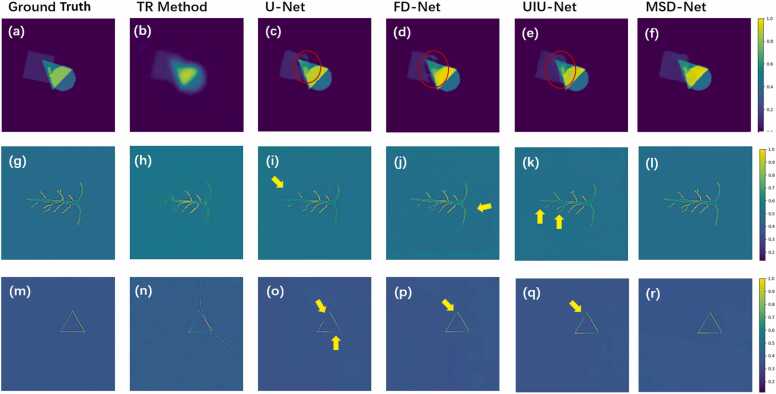
Reconstructed results of simulated dataset. The first column shows the ground truth of photoacoustic image, the second column displays the photoacoustic image reconstructed using the TR method. The third to fifth columns show the images reconstructed using U-Net, FD-Net, UIU-Net, and MSD-Net, respectively.

Fig. 4 shows the reconstruction results of the simulated dataset (including random targets, numerical blood vessel phantoms, and triangular targets) and compares the performance of the TR method, U-Net, FD-Net, UIU-Net, and MSD-Net. Firstly, the TR method shows poor reconstruction performance, with almost no texture features of the targets retained. As shown in Fig. 4(b), (h), and (n), although the target positions are accurately located and their shapes are roughly reconstructed, there are still significant artifacts, blurred edges, and loss of details. Secondly, when using U-Net for reconstruction, most of the artifacts are removed compared to the TR method, but some artifacts and details are still missing. For example, in Fig. 4(c), as indicated by the red circle, the rectangle and triangle are completely separated, but they should be overlapping in the ground truth. FD-Net also exhibits this issue, with UIU-Net performing slightly better. MSD-Net achieves the best reconstruction, clearly displaying the overlapping relationship of the targets and successfully restoring the small rectangle within the triangle. As shown in Fig. 4(i), (j), (k), and (l), indicated by the yellow arrows, U-Net and FD-Net mostly reconstruct the main structure of the blood vessel phantom, however, artifacts and background noise are still present. With the introduction of the UIU block, both UIU-Net and MSD-Net improve in reconstructing the main structure, with MSD-Net demonstrating a more significant advantage in detail restoration As shown in Fig. 4(o), (p), (q), and (r), indicated by the yellow arrows, in U-Net, FD-Net, and UIU-Net, there are gaps or cracks along the edges of the triangle, while MSD-Net removes most of the artifacts and accurately reconstructs the finer details, restoring the complete triangle. Additionally, it successfully reconstructs both the global edge features and local texture features of the object. This advantage may be attributed to the precise segmentation of the main structure by the UIU block and the superior detail restoration capability of the Dense Block.

### 4.3 Performance of MSD-net on *phantom and in vivo dataset*

**Fig. 5:**
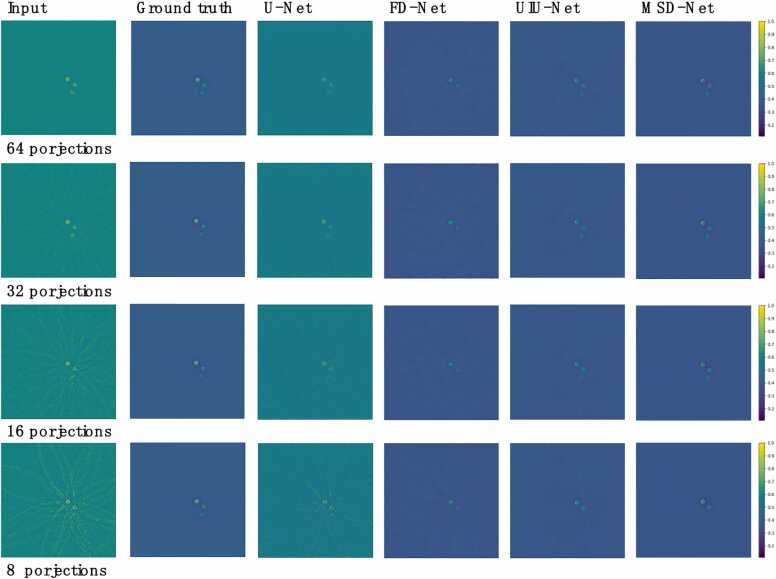
Phantom dataset reconstructed results (agar cylinders). The first column shows the photoacoustic images of the agar cylinder reconstructed using UBP with 8, 16, 32, and 64 projections, respectively. The second column shows the ground truth photoacoustic images of the agar cylinder using 512 projections. The third to fifth columns show the images reconstructed using U-Net, FD-Net, UIU-Net and MSD-Net, respectively.

On the phantom dataset(agar cylinder), the overall performance of U-Net is weak, especially when the number of projections is 8, there are a lot of artifacts and background noise in the reconstructed image, and the targets are almost unrecognizable. As the number of projections increases, the artifacts are reduced, but they are still obvious (as shown in the third column of Fig. 5). FD-Net has improved performance compared to U-Net and successfully removed some background noise and artifacts (as shown in the fourth column of Fig. 5). UIU-Net performs better than FD-Net, while MSD-Net performs best, being able to fully reconstruct three targets when the number of projections is 8, while other networks can only reconstruct two, and performs well in removing artifacts and background noise.

**Fig. 6:**
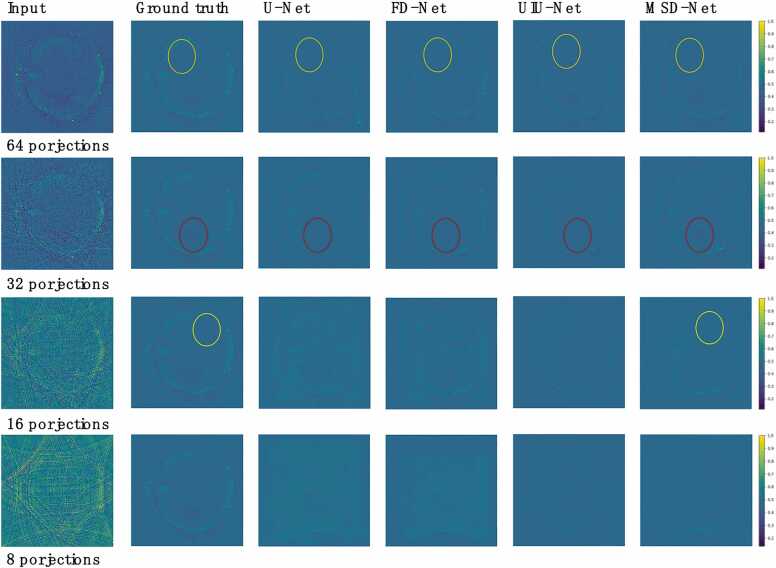
*in vivo* dataset reconstructed results (mice). The first column shows cross-sectional photoacoustic images of whole-body mice reconstructed using UBP with 8, 16, 32, and 64 projections, respectively. The second column shows the ground truth cross-sectional photoacoustic images of whole-body mice reconstructed using UBP with 512 projections. The third to fifth columns show images reconstructed using U-Net, FD-Net, UIU-Net and MSD-Net.

**Fig. 7:**
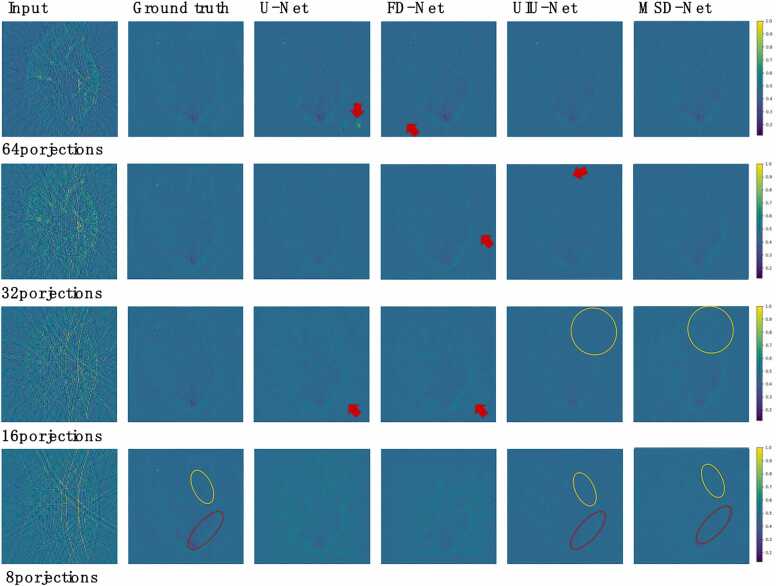
*in vivo* dataset reconstructed results. The first column shows cross-sectional photoacoustic images of whole-body mice reconstructed using UBP with 8, 16, 32, and 64 projections, respectively. The second column shows the ground truth cross-sectional photoacoustic images of whole-body mice reconstructed using UBP with 512 projections. The third to fifth columns show images reconstructed using U-Net, FD-Net, UIU-Net, and MSD-Net.

For the *in vivo* dataset, when the number of projections is 8, the reconstructed images of U-Net and FD-Net both have a lot of artifacts, the target contour and details are seriously lost, and the image is blurred. However, UIU-Net significantly reduces most of the artifacts, basically restores the target contour, and the reconstruction quality is significantly better than U-Net and FD-Net. MSD-Net performs even better, not only further reducing artifacts, but also performing well in preserving the details of the target area, and clearly reconstructing the boundaries and shapes of the target. When the number of projections is low, MSD-Net effectively retain more original information, as shown in the yellow and red circles in Fig. 6 and Fig. 7, demonstrating its powerful reconstruction capabilities under low-projections.

### 4.4 Ablation study

**Fig. 8:**
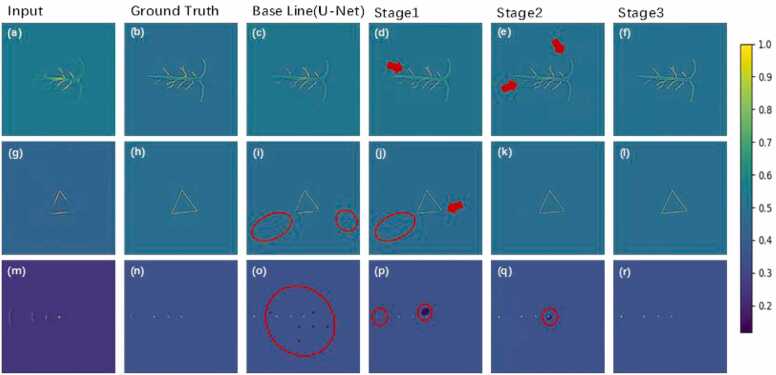
Ablation study results on the simulated dataset. The first column shows the input images, the second column displays the ground truth images, the third column presents results from the baseline U-Net, the fourth column corresponds to Stage 1 (U-Net + UIU Block), the fifth column represents Stage 2 (U-Net + UIU Block + Dense Blocks), and the sixth column shows Stage 3 (U-Net + UIU Block + Dense Blocks + Multi-Scale Connections).

To evaluate the impact of various components on the performance of the MSD-Net model, we designed and conducted ablation experiments. Specifically, we gradually replaced different components in the model and observed their effects on image reconstruction quality. The baseline network architecture was U-Net, and the experiments included the following configurations: Stage 1) adding the UIU block to the encoding path; Stage 2) adding dense blocks to the decoding path; Stage 3) introducing multi-scale connections to the encoding path. For the traditional U-Net network, the SSIM and PSNR of the reconstructed images were 0.9547 and 33.74 dB respectively. As shown in Fig. 8(c), the reconstructed images contained significant background noise and artifacts, and the edges of the target object were unclear. In Fig. 8(i), a small amount of artifacts remained, and Fig. 8(o) showed black noise spots. After adding the UIU block, the SSIM and PSNR improved to 0.9728 and 34.55 dB. As shown in Fig. 8(d), the main structure of the reconstructed image gradually became clearer, and much of the background noise was removed, although there was still room for improvement in the details. Fig. 8(j) still contained some artifacts, and black noise spots were still visible in Fig. 8(p). Subsequently, after adding dense blocks to the decoding path, the SSIM and PSNR further improved to 0.9736 and 35.34 dB. As seen in Fig. 8(e), the main structure of the reconstructed image became very clear, with almost all background noise removed. The triangular shape reconstructed in Fig. 8(k) was nearly fully restored, and the black noise spots in Fig. 8(q) were significantly reduced. Finally, after adding the multi-scale connection module to the end of the decoding path, the SSIM and PSNR reached 0.9838 and 39.82 dB. As shown in Fig. 8(f), (l), and (r), all images were accurately reconstructed, with almost all artifacts removed and details well restored. The multi-scale connection module significantly improved the quality of image reconstruction. Therefore, the UIU block, dense blocks, and multi-scale connections have all been proven effective in our proposed architecture.

## 5 Conclusion and discussion

In this work, we propose a network called MSD-Net to introduce multi-scale dense connections into photoacoustic reconstruction algorithms. The network is able to reconstruct 2D PAT images from sparse data, significantly removing artifacts and improving the performance of photoacoustic reconstruction algorithms. We trained, tested, and evaluated MSD-Net on simulation datasets, phantom datasets, and *in vivo* datasets, and compared it with U-Net, FD-Net, and UIU-Net. Experimental results show that MSD-Net performs well in all indicators and is significantly better than other methods, fully verifying its superior performance under sparse data conditions. Additionally, MSD-Net has improved performance while reducing memory usage.

However, there is still room for improvement in the efficiency and reliability of MSD-Net. Firstly, in terms of skip connections, we currently only use the traditional U-Net concatenate convolution without further improvements. In future work, we plan to explore other multi-scale skip connections or skip connection methods similar to UNet3 + and UNet+ + to find more suitable network architectures for photoacoustic reconstruction.

As a post-processing method, MSD-Net is still influenced by standard methods. Its performance is heavily dependent on the quality of TR reconstruction. If image features are occluded by severe artifacts or missing after TR reconstruction, these features are likely to be incorrectly reconstructed or entirely lost during CNN reconstruction. Information loss occurs due to sparse sampling, and the initial step of reconstructing images directly from sensor data also discards potentially useful information and introduces artifacts. Utilizing a direct reconstruction algorithm could potentially enhance reconstruction speed while simultaneously mitigating information loss, thereby preserving more details of the mouse brain.

Regarding datasets, although photoacoustic imaging is not yet widely used in clinical practice and there are few public datasets available, the quality of datasets is crucial to network performance. To reduce dependence on labeled data, we are exploring the introduction of unsupervised learning methods, which will be one of our future research priorities.

## CRediT authorship contribution statement

**Yiming Qian:** Writing – review & editing, Validation. **Yichao Meng:** Writing – review & editing, Supervision, Resources, Conceptualization. **Liangjie Wang:** Writing – review & editing, Writing – original draft, Visualization, Validation, Software, Methodology, Investigation, Data curation.

## Declaration of Competing Interest

The authors declare that they have no known competing financial interests or personal relationships that could have appeared to influence the work reported in this paper.

## Data Availability

Data will be made available on request.
